# Digestive Tract Morphology and Gut Microbiota Jointly Determine an Efficient Digestive Strategy in Subterranean Rodents: Plateau Zokor

**DOI:** 10.3390/ani12162155

**Published:** 2022-08-22

**Authors:** Shou-Dong Zhang, Gong-Hua Lin, Ji-Ru Han, Yu-Wei Lin, Feng-Qing Wang, De-Chen Lu, Jiu-Xiang Xie, Jin-Xin Zhao

**Affiliations:** 1 Ministry of Education Key Laboratory for Biodiversity Science and Ecological Engineering, Coastal Ecosystems Research Station of the Yangtze River Estuary, Shanghai Institute of Eco-Chongming (SIEC), Fudan University, Shanghai 200433, China; 2 Key Laboratory of Adaptation and Evolution of Plateau Biota, Northwest Institute of Plateau Biology, Chinese Academy of Sciences, Xining 810008, China; 3 School of Life Sciences, Jinggangshan University, Ji’an 343009, China; 4 Population Health and Immunity Division, The Walter and Eliza Hall Institute of Medical Research, Parkville, VIC 3052, Australia; 5 Infection Program, Department of Microbiology, Biomedicine Discovery Institute, Monash University, 19 Innovation Walk, Clayton, VIC 3800, Australia; 6 Shandong Marine Resource and Environment Research Institute, Yantai 264006, China; 7 College of Marine Science, Shandong University at Weihai, Weihai 264209, China; 8 State Key Laboratory of Plateau Ecology and Agriculture, College of Agriculture and Animal Husbandry, Qinghai University, Xining 810016, China

**Keywords:** subterranean, plateau zokor, *Myospalax baileyi*, digestive tract morphology, gut microbiota, digestion strategy, crude fiber

## Abstract

**Simple Summary:**

Investigation of mechanistic insights of digestive strategies in rodents can be difficult, but it is important to understand how rodents adapt to different environments. Applying physiological analyses to compare the differences between digestive tracts in plateau zokor and laboratory rats, we found that the length and weight of the digestive tract of the plateau zokor was significantly greater than the laboratory rat. Particularly, the weight and length of the large intestine and cecum in plateau zokor is three times that of the laboratory rat. Our gut microbiota analysis results showed that bacteria associated with cellulose degradation were significantly enriched in laboratory rats, when compared to plateau zokor. However, both plateau zokor and laboratory rats were predicted to share the same functions in carbohydrate metabolism and energy metabolism. Our findings suggest that both the morphology of the digestive tract and gut microbiota are vital to the digestion in wild rodents.

**Abstract:**

Rodents’ lifestyles vary in different environments, and to adapt to various lifestyles specific digestion strategies have been developed. Among these strategies, the morphology of the digestive tracts and the gut microbiota are considered to play the most important roles in such adaptations. However, how subterranean rodents adapt to extreme environments through regulating gut microbial diversity and morphology of the digestive tract has yet to be fully studied. Here, we conducted the comparisons of the gastrointestinal morphology, food intake, food assimilation, food digestibility and gut microbiota of plateau zokor *Eospalax baileyi* in Qinghai-Tibet Plateau and laboratory rats *Rattus norvegicus* to further understand the survival strategy in a typical subterranean rodent species endemic to the Qinghai-Tibet Plateau. Our results revealed that plateau zokor evolved an efficient foraging strategy with low food intake, high food digestibility, and ultimately achieved a similar amount of food assimilation to laboratory rats. The length and weight of the digestive tract of the plateau zokor was significantly higher than the laboratory rat. Particularly, the weight and length of the large intestine and cecum in plateau zokor is three times greater than that of the laboratory rat. Microbiome analysis showed that genus (i.e., *Prevotella*, *Oscillospira*, CF231, *Ruminococcus* and *Bacteroides*), which are usually associated with cellulose degradation, were significantly enriched in laboratory rats, compared to plateau zokor. However, prediction of metagenomic function revealed that both plateau zokor and laboratory rats shared the same functions in carbohydrate metabolism and energy metabolism. The higher digestibility of crude fiber in plateau zokor was mainly driven by the sizes of cecum and cecum tract, as well as those gut microbiota which associated with cellulose degradation. Altogether, our results highlight that both gut microbiota and the morphology of the digestive tract are vital to the digestion in wild rodents.

## 1. Introduction

To cope with environmental pressure, different animals have formed their own digestive strategies [1,2]. The efficiency of accessing and processing food is essential to wild animals’ survival and reproduction [3]. The structure of the digestive tract has been shown to play a key role in the digestive strategies in animals, as the digestive tract is the most important place for food digestion and absorption. The morphology and structure of the digestive tract are closely related to diet, prey quality and energy requirements, and affect the efficiency of food absorption. The main functions of digestive tracts are digesting and absorbing food [4,5,6]. Various diets could cause significant differences in the morphology of the digestive tract. For example, Schieck and Millar (1985) compared the relationship between the digestive tract and diet in 35 mammals and found that the length of the digestive tract in different animal groups was: herbivorouse > omnivorous > carnivorous [7]. Perrin and Curtis (1980) compared the relationship between the digestive system morphology and feeding habits in 19 rodents species in South Africa, and proposed that feeding habit is one of the main reasons for the difference in digestive tract morphology across species [8]. Moreover, Gross et al. (1985) experimented on *Microtus ochrogaster* in the laboratory, and confirmed that the combination of low temperature and high-fiber foods caused significant increases in length and dry mass of total digestive tract [4].

Gut microbiota also plays a crucial role in food digestion [9] and energy balance [10] for the host. Gut microbiota, as a dynamic ecosystem, has been shown to be temporarily stable in the changeless setting [11]. Additionally they may change dynamically following multiple factors (e.g., time, environment and host) [12]. The details of these factors include the season, diet, host genotype, age, sex, and host healthy condition (e.g., metabolic syndrome, inflammatory bowel diseases and obesity) [13,14,15,16]. Among these factors, diet has been considered as one of the most important determinants to gut microbiota composition [15,17,18]. There are significant differences in the composition of microbiomes in herbivorous, omnivorous and carnivorous animals, with herbivorous animals having the highest gut microbiota diversity [19]. Specific diets (e.g., whole grains, fruits, nuts, vegetables, beans) may affect the composition of the gut microbiota [20,21]. Whole grain foods contain high levels of dietary fiber in general. However, mammals do not have the enzyme that can directly degrade fibers. Therefore, these fibers are supposed to be digested by microbiome communities in cecum [22], and affect the composition of gut microbiota in the meantime [12].

More recently, subterranean rodents received extensive attention with their special underground lifestyle [23,24]. Previous studies showed that underground life history characteristics resulted in high energy expenditure [25,26]. Studies have shown that the Namib Desert golden moles *Eremitalpa namibensis* moving in the solid soil consume 260 times more than when moving the same distance on the ground [27]. Subterranean rodents consume huge amounts of energy to forage underground, which makes subterranean rodents an ideal group to study for foraging and digestion strategies.

Plateau zokor (*Eospalax baileyi*) is a solitary and entirely subterranean burrowing endemic rodent species, inhabiting areas of 2600 to 4600 m above sea level on Qinghai-Tibet Plateau [23,24]. Plateau zokor spend most their life in underground nests and foraging, and burrowing activity mainly takes place at a depth of 3–20 cm under the ground [28]. This underground lifestyle means that plateau zokor must excavate for courtship and foraging [25,26]. Previous studies showed that the amount of soil excavated and launched to the ground by one individual can reach one tonne every year. This process suggested a huge energy consumption [27] and to search for food, plateau zokor have to consume a considerable amount of energy. Studies found that plateau zokor prefer ingesting the higher nutrition part of plants, such as roots and tubers [29,30]. However, there is no report about the digestive strategies in plateau zokor.

The present study aims to investigate the digestive strategies in plateau zokors by comparing the food intake, assimilation, and digestibility of subterranean rodent plateau zokors with the similar size laboratory rats (*Rattus norvegicus*). The study also explores the main causes of the different digestive strategies in both plateau zokors and laboratory rats by comparing the morphology of the digestive tract and the composition of the gut microbiota.

## 2. Materials and Methods

### 2.1. Sample Collection

Zokors were captured used a novel ground arrow [24] during May 2012 and 2013 in Dongxia Town, Datong County (37°03′ N, 101°47′ E, 2994 m above sea level), Qinghai, China. After being transferred back to the lab, we maintained them individually in iron cages (35 × 25 × 20 cm) with wood shavings in a constant environment (at 25 ± 1 °C, length of day: 14 h). All the captured zokors passed the health examination to prove that they were healthy. We introduced zokors to artificial feed (purchased from Beijing Keao Xieli Feed Co., Ltd., Beijing, China) in the Key Laboratory of Adaptation and Evolution of Plateau Biota, Northwest Institute of Plateau Biology, Chinese Academy of Sciences for at least one year. The water intake of the plateau zokor mainly depends on food in the wild. In the laboratory, we use water bottles to supply plenty of water for zokors. A total of 12 laboratory rats (six males and six females) were purchased from the Experimental Animal Center of Gansu University of Chinese Medicine in August 2014. Rats were fed in the same way as zokors.

### 2.2. Food Consumption and Digestibility

The experiment was conducted in September 2014 in the same lab. We recorded the body mass of the 12 plateau zokors (six males and six females) and 12 laboratory rats (six males and six females) (Table S1) and then put them individually into plastic cages (35 × 25 × 20 cm) where the standard polypropylene bottoms were replaced with iron mesh; this maintained the feed pellets while allowing the feces to drop down [24]. In the afternoon (18:00), 50 g of feed pellets was provided to each zokor and rat, and the next afternoon, the remaining feed was replaced by another 50 g of pellets (nutritional composition see Table S2); this was supplemented with sufficient water, and the remaining feed and feces from the previous day were removed and replaced by another 50 g of feed pellets every day. After an adaptive feeding for one month, we maintained each animal in a plastic box that was wiped and dried with 75% ethanol. Lifting their tails, they will discharge the feces. We collected a total 24 feces samples and stored them at −40 °C. After that, the same feeding method was adopted. The remaining feed and feces from the previous day were picked out every day, dried in an oven at 60 °C to constant weight, and later weighed by an electronic analytical balance (0.0001 g, Metter Toledo Inc., Columbus, OH, USA). The test lasted for 7 days, and the feces of each test animal were mixed and stored.

To determine the digestibility of protein and crude fiber of zokors and rats, we need to measure the protein and crude fiber content in feed and feces. A total of 12 portions of feces of plateau zokor, 12 portions of feces of rats and 6 portions of feed were ground into powder for the determination. The protein content was determined by the KjeltecTM 8400 Kjeldahl nitrogen analyzer (Shanghai Fiber Inspection Instrument Co., Ltd., Shanghai, China) [24]; the crude fiber content was determined by the crude fiber analyzer (Huaye SLQ-6, Shanghai Fiber Inspection Instrument Co., Ltd., Shanghai, China) [24].

### 2.3. Digestive Tract Morphology

After the trial, the zokors and rats were killed and the lengths of small intestines, large intestines and caecum were measured. The small intestines, large intestines, and caecum were emptied and washed with normal saline and dried at 60 °C to constant weight and later weighed by an electronic analytical balance (0.0001 g, Metter Toledo Inc.) [24]. All animal procedures in this study were conducted with ethical approval from the Ethics Committee, Northwest Institute of Plateau Biology, Chinese Academy of Sciences (No. NWIPB–2014–01). No specific permission was required for activities in this study.

### 2.4. DNA Extraction and MiSeq Sequencing of 16S rRNA Gene Amplicons

DNA extraction from 23 feces (~0.3 g, Z4 lost) was extracted using Ezup genomic DNA extraction kit for soil (Sangon Biotech, Shanghai, China, Cat# SK8264). DNA concentration and quality were checked using a NanoDrop Spectrophotometer (Thermo Scientific, IL, Waltham, MA, USA). Extracted DNA was diluted to 10 ng/μL and stored at −40 °C for downstream use.

The universal primer 515F (5′-GTGCCAGCMGCCGCGGTAA-3′) and 806R (5′-GGACTACHVGGGTWTCTAAT-3′) with 12 ntunique barcode was used to amplify the V4 hypervariable region of 16S rRNA gene for pyrosequencing using Miseq sequencer [31,32]. The ITS3_KYO2F/ITS4R primer pair was used to amplify the eukaryotes from feces DNAs [33]. The procedures of PCR amplification, gel extraction, and Miseq sequencing followed the methods of Li et al., 2016 [34].

### 2.5. Bioinformatics Analysis

The sequence data were processed using QIIME Pipeline–Version 1.7.0 (http://qiime.org/, accessed on 25 June 2022). All sequence reads were trimmed and assigned to each sample based on their barcodes. The sequences with high quality (length > 150 bp, without ambiguous base ‘N’, and average base quality score > 30) were used for downstream analysis [35].

For the analysis of 16S rRNA gene sequences, chloroplasts from our large sequencing data sets were removed using the metaxa2 software tool [36]. The aligned 16S rRNA gene sequences were used for chimera check using the Uchime algorithm [37]. Sequences were clustered into operational taxonomic units (OTUs) at a 97% identity threshold using CD-HIT-EST [38]. Those sequences not classifying to bacteria (Eukaryota and Archaea lineages) were removed [12]. Singleton sequences were also filtered out [12]. To standardize sampling efforts across samples, all the samples were randomly resampled to 6679 reads.

We calculated alpha-diversity (Goods coverage, the chao1 estimator of richness, Shannon’s diversity index, Simpson’s diversity index and observed OTUs) and beta-diversity (PCoA, UniFrac) analyses through the QIIME pipeline, for which the rarefaction curves were generated from the observed species [37]. Taxonomy was assigned using the Ribosomal Database Project classifier [39]. PCA analysis (packages “ade4”and “ggplot2”) [40,41], Anosim analysis (packages “vegan,” anosim function) [41,42], heatmap (packages “pheatmap”) [43] and Metastats analysis were performed with R software (packages “optparse”) [41,44]. LefSe software was used to analyze LefSe (linear discriminant analysis effect size) analysis [45]. The LDA (linear discriminant analysis) score was 4 [41].

### 2.6. Statistical Analysis

Based on dry matter of provided feed (PF), remaining feed (RF), and feces output (FO), we calculated the following indices: food intake (FI) = PF − RF; food assimilation (FA) = FI − FO; food digestibility (FD) = FA/FI × 100% [24]. Consequently, combined with protein content of feed (P1%) and feces (P2%), and crude fiber content of feed (C1%) and feces (C2%), we calculated the following indices: protein assimilation (PA) = FI × P1% − FO × P2%, protein digestibility (PD) = PA/(FI × P1%) [24], crude fiber assimilation (CA) = FI × C1% − FO × C2%, and crude fiber digestibility (CD) = PA/(FI × C1%)

In order to improve the statistical reliability and also to exclude the effects of body weight, we used the General Linear Mode to test the difference between zokors and rats in FI, FO, FA, FD, PA, PD, CA, CD, the dry mass and the length of small intestines, large intestines and caecum, with the body mass as the covariate.

The difference statistical analysis of relative abundance of target gene copies for specific species (% of total bacterial 16S rRNA gene) between plateau zokors and laboratory rats at Phylum, Class, Order, Family and Genus level and alpha-diversity (Goods coverage, chao1 estimator of richness, Shannon’s diversity index, Simpson’s diversity index and observed OTUs) were compared using Analysis of Variance (ANOVA). The core microbiota of plateau zokor and laboratory rats was defined as those genera recovered from all individuals in each species. Spearman’s co-related test conducted in R was used to assess the synergistic relationship of core genus of plateau zokor and laboratory rats. The significance level was set at 0.05. All statistical analyses were carried out in SPSS 20.0.

### 2.7. Predicted Metagenomes

PICRUSTv1.0.0 [46] was used to predict abundances of KOs from OTU abundances rarefied at 6679 reads per sample. We only focused on the gene functions associated with metabolism. Two-tailed t-tests (Bonferroni corrected) were performed to test the differences in gene functions between plateau zokors and laboratory rats.

## 3. Result

### 3.1. Food Consumption and Digestibility

**Figure 1:**
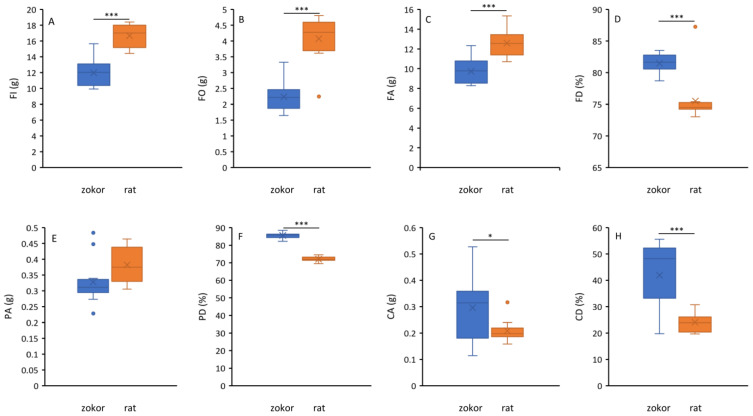
The difference between plateau zokors and laboratory rats regarding daily food intake (FI, (**A**)), daily feces output (FO, (**B**)), daily food assimilation (FA, (**C**)), food digestibility (FD, (**D**)), daily protein assimilation (PA, (**E**)), protein digestibility (PD, (**F**)), daily crude fiber assimilation (CA, (**G**)) and crude fiber digestibility (CD, (**H**)). The asterisks refer to significant difference (* means *p* < 0.05, *** means *p* < 0.001).

The daily food intake (zokor: 11.97 ± 1.72 g, rat: 16.66 ± 1.45 g), daily feces output (zokor: 2.23 ± 0.47 g, rat: 4.08 ± 0.71 g) and daily food assimilation (zokor: 9.74 ± 1.28 g, rat: 12.59 ± 1.34) of plateau zokor were significantly (*F* > 28.25, *p* < 0.001) lower than laboratory rats (Figure 1A–C), and the food digestibility (zokor: 81.47 ± 1.58, rat: 74.50 ± 3.76), protein digestibility (zokor: 85.58 ± 1.63, rat: 72.06 ± 1.56) and crude fiber digestibility (zokor: 41.92 ± 12.71, rat: 24.11 ± 3.76) of plateau zokor were significantly (*F* > 21.65, *p* < 0.001) higher than laboratory rats (Figure 1D,F,H). The daily crude fiber assimilation of plateau zokors (0.29 ± 0.12) were significantly (*F* = 5.47, *p* = 0.03) higher than laboratory rats (0.21 ± 0.04), while the daily protein assimilation of plateau zokors (0.33 ± 0.07) have no significant difference (*F* = 4.26, *p* > 0.05) with laboratory rats (0.38 ± 0.06). The crude fiber digestibility of plateau zokor was nearly twice that of laboratory rats.

### 3.2. Digestive Tract Morphology

**Figure 2:**
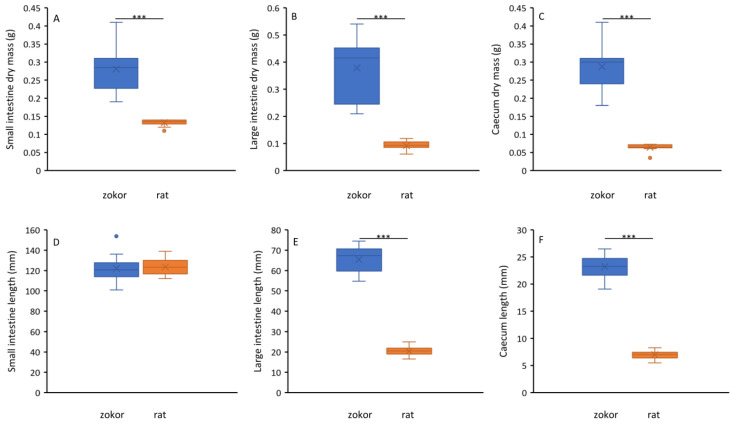
The difference between plateau zokors and laboratory rats about small intestine day mass (**A**), large intestine day mass (**B**), cecum dry mass (**C**), small intestine length (**D**), large intestine length (**E**) and cecum length (**F**). The asterisks refer to significant difference (*** means *p* < 0.001).

Except for the length of the small intestine, plateau zokors and rats have significant differences in the dry mass of the small intestine, large intestine and cecum, and length of the large intestine and cecum (*F* > 75.19, *p* < 0.001, Figure 2). The dry mass of the small intestine of the plateau zokor (0.28 ± 0.06 g) is twice that of the rat (0.13 ± 0.01 g); the dry mass of the large intestine (zokor: 0.38 ± 0.11 g, rats: 0.09 ± 0.02 g) and cecum (zokor: 0.28 ± 0.06 g, rats: 0.06 ± 0.01 g), and the length of the large intestine (zokor: 65.37 ± 6.50 cm, rats: 20.28 ± 2.33 cm) and cecum (zokor: 23.23 ± 2.25 cm, rats: 7.01 ± 0.77 cm) of the plateau zokor were more than three times that of the rats. The dry mass of the large intestine and cecum of the plateau zokor accounted for 46% of the entire digestive tract, while the dry mass of the large intestine and cecum of the rat accounted for only 32% of the entire digestive tract (Figure 2).

### 3.3. Gut Microbiota

**Figure 3:**
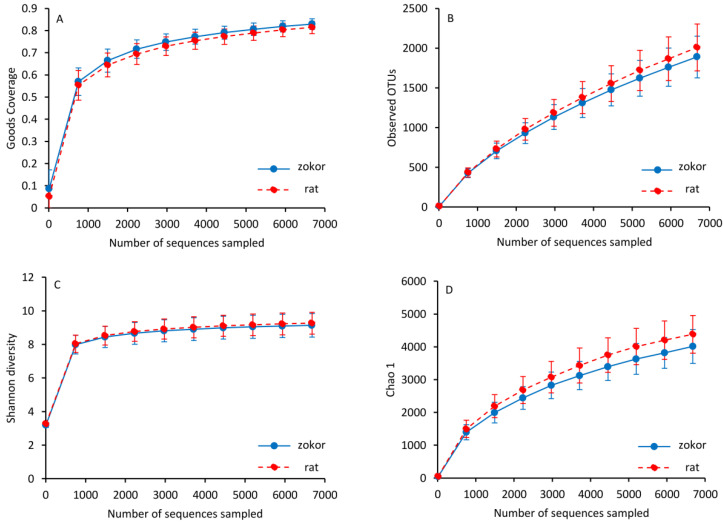
OTU-level rarefaction curves of (**A**) Goods coverage, (**B**) observed OTUs, (**C**) Shannon diversity and (**D**) Chao 1 between host species (plateau zokors/laboratory rats) across 23 samples.

A total of 1,070,323 sequences were obtained from 23 samples by 16S rRNA gene sequencing. When filtering out low-quality sequences, chloroplasts, OTUs containing only one sequence, and those sequences not belonging to bacteria, we obtained 942,902 effective sequences. For comparison, each sample was normalized to 6679 sequences. Clustering was performed with 97% sequence similarity, and a total of 34,614 different OTUs were identified. OTU-level rarefaction curves of Goods coverage and observed OTUs (or species) are nearly flat in different species (Figure 3A,B, Table 1), showing that most of the intestinal microbial diversity of plateau zokor and laboratory rats has been captured, indicating that the additional sequencing adds some rare groups.

**Figure 4:**
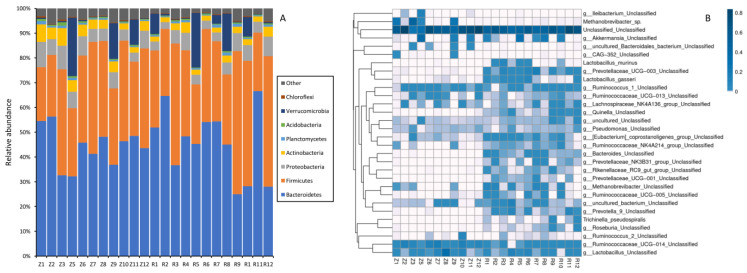
The compositions of host species (plateau zokors/laboratory rats) gut microbiota at phylum level across 23 samples (only top eight phyla with mean relative abundance > 0.1% were shown, (**A**)), and the heat maps plotted for the distribution of the species in each sample on different levels of classification (**B**).

Across all the samples, 96.1% of the total sequences of plateau zokor and 97.2% of the total sequences of laboratory rats were assigned into 31 phyla. The top eight phyla of plateau zokor and laboratory rats with mean relative abundance > 0.1% were shown in Figure 4A. *Bacteroidetes* (41.1 ± 9.8%), *Firmicutes* (31.7 ± 8.3%), *Proteobacteria* (6.0 ± 1.5%), *Verrucomicrobia* (4.2 ± 7.3%), *Actinobacteria* (3.9 ± 1.1%) were the five most dominant bacterial phyla in plateau zokor feces; other low-abundance (mean relative abundance < 1%) phyla included *Planctomycetes*, *Acidobacteria*, *Chloroflexi*, *Tenericutes*, TM7, *Gemmatimonadetes* (Figure 4A, Table 2). Five most dominant bacterial phyla in laboratory rats’ feces were *Bacteroidetes* (44.1 ± 13.4%), *Firmicutes* (36.1 ± 11.7%), *Proteobacteria* (4.9 ± 1.7%), *Verrucomicrobia* (4.8 ± 7.0%), *Actinobacteria* (2.5 ± 0.8%); other low-abundance (mean relative abundance < 1%) phyla included *Planctomycetes*, *Acidobacteria*, *Chloroflexi*, *Crenarchaeota*, *Tenericutes*, TM7, *Gemmatimonadetes* (Figure 4A, Table 2). The relative abundance of unclassified bacteria in plateau zokor and Laboratory rat feces were 3.9% and 2.8%, respectively, indicating that there should be some novel bacterial taxa in plateau zokor and laboratory rat feces. At genus level, *Akkermansia* (4.0 ± 7.3%), *Lactobacillus* (3.7 ± 5.6%), *Ruminococcus* (2.8 ± 1.8%), *Prevotella* (1.3 ± 1.6%) were the four most dominant bacterial genera in plateau zokor feces, while *Prevotella* (20.6 ± 14.8%), *Akkermansia* (4.5 ± 6.9%), *Lactobacillus* (4.5 ± 3.5%), CF231 (2.8 ± 2.6%), *Ruminococcus* (2.1 ± 0.2%), *Oscillospira* (2.0 ± 1.3%), *Bacteroides* (1.2 ± 0.9%) were the seven most dominant bacterial genera in laboratory rat feces (Figure 4B, Table 3).

The difference in gut microbiota composition between plateau zokor and laboratory rat feces were compared in phylum, (Table 2), genus (Table 3), class (Table S3), order (Table S4) and family (Table S5) levels. The dominant phylum *Bacteroidetes*, *Firmicutes*, *Proteobacteria* and *Verrucomicrobia* were similar in plateau zokor and laboratory rat feces, while *Actinobacteria* were more abundant in plateau zokor and *Tenericutes* were more abundant in laboratory rats (Table 2). At the genus level, the relative abundance of *Prevotella*, *Oscillospira*, CF231, *Ruminococcus*, and *Bacteroides* was significantly enriched in laboratory rats (16-fold, 3-fold, 20-fold, 6-fold, and 68-fold than in Plateau zokor, respectively), while *Altererythrobacter*, and *Pseudomonas* were more abundant in plateau zokor.

**Figure 5:**
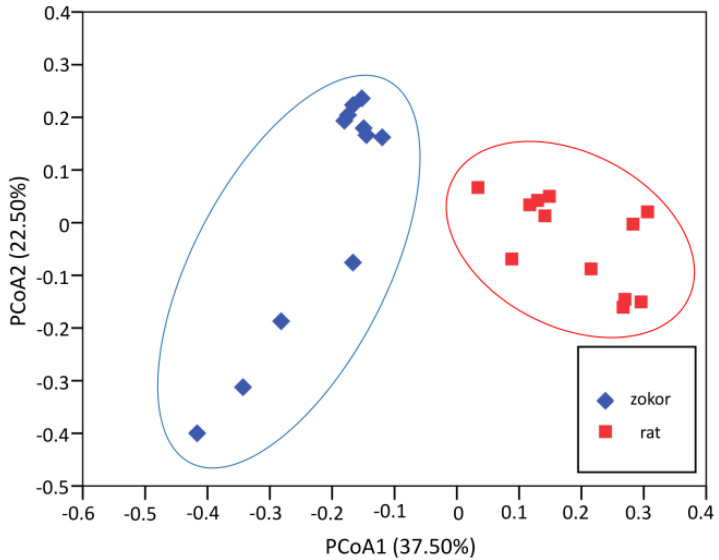
Principal coordinate analysis of gut microbial communities between host species (plateau zokors/laboratory rats) based on weighted UniFrac distance.

Alpha diversity analysis did not show any statistical difference in Goods coverage, the chao1 estimator of richness, Shannon’s diversity index, Simpson’s diversity index and observed OTUs between plateau zokors and laboratory rats (*F* < 2.62, *p* > 0.12; Table 1, Figure 3). Meanwhile, beta diversity analysis showed that gut microbiota structure was structurally segregated between plateau zokors and laboratory rats, based on weighted UniFrac distance (Anosim analysis, *R* = 0.23, *p* = 0.002) (Figure 5).

**Figure 6:**
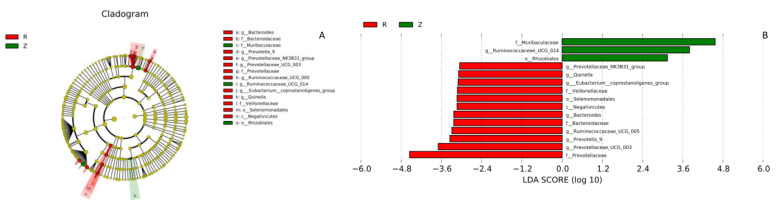
Lefse analysis of the gut microbiota in plateau zokors (the abbreviations Z)/laboratory rats (the abbreviations R). (**A**) Cladogram of gut microbiota communities. (**B**) Biomarker genes and their LDA scores (LDA score = 4).

According to Lefse analysis (Figure 6), 15 biomarkers were identified (LDA score: 4). The relative abundance of *Ruminococcaceae_UCG-014* was significantly higher in plateau zokors, whereas *Prevotellaceae_NK3B31_group*, *Quinella*, *Eubacterium_coprostanoligenes_group*, *Bacteroides*, *Ruminococcaceae_UCG-005*, *Prevotella_9*, *Prevotellaceae_UCG-003* were significantly higher in laboratory rats (Figure 6).

**Figure 7:**
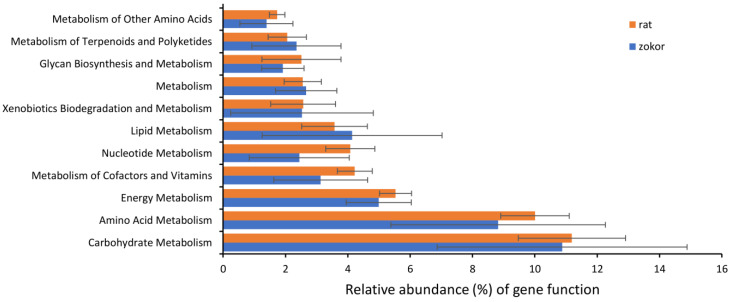
Results of PICRUSt metagenome prediction showing the differences in predicted gene relative abundance of KEGG orthology groups between host species (plateau zokors/laboratory rats).

PICRUSt gene function prediction analysis identified differences in 11 metabolic-related pathways (Figure 7). There was no significant difference in the relative abundance of these 11 metabolic-related pathways (amino acid metabolism, carbohydrate metabolism, energy metabolism, glycan biosynthesis and metabolism, lipid metabolism, metabolism of cofactors and vitamins, metabolism of other amino acids, metabolism of terpenoids and polyketides, nucleotide metabolism, xenobiotics biodegradation and metabolism) between plateau zokor and laboratory rats (*F* < 2.23, *p* > 0.05, Figure 7).

## 4. Discussion

Animals within different environmental pressures could form their own different digestive strategies. The unique underground lifestyle of the subterranean rodents determines high energy expenditure in their life history such as digging format, foraging and other activities [25,26]. For foraging, the energy consumption is enormous, so they need to have a more efficient diet digestibility to buffer the energy expenditure [27,28]. Plateau zokor, a typical subterranean rodent, is accompanied by excavation activities and consumes more energy compared with ground rodents [27,28]. Therefore, even with less food intake, plateau zokors can still achieve the same assimilation to the underground living environment with the similar size rat by their higher digestibility. However, the causes of such high efficiency of digestive strategies in plateau zokor remains unclear. Mammals not only digest food by utilizing the digestive enzymes that are produced by the digestive tract, but also rely on the decomposition of gut microbes.

The morphological structure of the digestive tract is highly correlated to energy requirements, and its morphology indicates the energy pressure faced by small herbivorous mammals in the wild [47]. The small intestine is the main organ for the digestion and absorption of nutrients, and changes in the small intestine reflect the energy requirements in animals [48,49]. There is no difference in the length of the small intestine between the plateau zokor and the laboratory rat, but the dry mass of the small intestine in the plateau zokor is significantly larger than the lab rat. The difference in the dry mass is due to more microvilli on the inner wall and the thicker mucosa of the small intestine of the plateau zokor [24], which is supposed to be the main reason for the food digestibility in plateau zokors. The cecum and large intestine are the main fermentation sites in hindgut fermenting animals, which are more sensitive to the cellulose content in food [48,49]. The increased capacity of the cecum and large intestine can prolong the residence time of digestive materials in the digestive tract, thereby improving the efficiency of digestion of the cellulose [48,49]. The evolved large intestine and cecum in plateau zokor may be one of the reasons for the high crude fiber digestibility. In addition, rats are usually fed on crop seeds, fruits, and animal foods in the wild. All these foods contain low crude fiber contents, while plateau zokor mainly fed on plant rhizomes which have a high crude fiber content [29]. Therefore, the plateau zokor has a more developed large intestine and cecum, which reflects its adaptability to its special life characteristics in terms of the morphology and function of the digestive organs.

With the increasing development of next-generation sequencing techniques, gut microbiota has been increasingly studied. Gut microbiota has been proposed to play an important role in the digestion and absorption in animals [9,12]. Plateau zokor has a very high utilization efficiency of crude fiber, and the crude fiber is usually divided into arabinoxylans, xyloglucans, b-glucans, glucomannans, and galactomannans by enzymes in the intestine [50]. Although many herbivores are capable of digesting cellulose, there are no digestive cellulose coding genes being reported in the mammalian genome [22]. This suggests that the digestion of cellulose by mammals fertilized by the hindgut mainly depends on the action of the gut microbiota. For example, a variety of glycoside hydrolase-encoding genes including cellulase and glucosidase were found by analyzing the metagenome of giant panda gut microbiota [22]. Many members of genera such as *Prevotella*, *Bacteroides*, *Ruminococcus* have been considered to be the essential groups for hydrolyzing cellulose [51,52,53,54]. Gut microbiota relies on these recalcitrant fibers for producing energy [55]. In addition, diet has been identified to shape the diversity, structure, and function of the mammalian gut microbiota [19,56,57]. In the present study, we investigate the difference in the gut microbiota between the plateau zokors and laboratory rats under the same food feeding condition, to further understand the effects of gut microbiota on the high digestibility of crude fiber in the plateau zokors.

In this study, the quality of sequencing results was high by OTU-level rarefaction curves of Goods coverage, the chao1 estimator of richness, Shannon’s diversity index, and observed OTUs. The species coverage of bacteria in the feces of plateau zokors and laboratory rats has reached about 80%, and the sample sequencing data are reasonable, which can better reflect the gut microbiota in animal feces. Therefore, it is feasible to compare the gut microbiota community between plateau zokors and laboratory rats. The dominant phyla *Bacteroidetes* and *Firmicutes* in the plateau zokors’ and laboratory rats’ guts accounted for 72% and 80%, respectively, of the 16S rRNA gene sequences, which was consistent with previous research [58,59]. The *Bacteroidetes* and *Firmicutes* are mainly responsible for diet decomposition [60], and there is a high degree of similarity in mammals.

At Phyla level, the dominant phylum *Actinobacteria* were more abundant in plateau zokor, while the core phylum *Tenericutes* were more abundant in laboratory rats. Turnbaugh et al. (2009) reported that the increased number of *Actinobacteria* was strictly related to obesity, and 75% of obese-rich genes (involving carbohydrate, lipid and amino acid metabolism) are derived from *Actinobacteria* [17]. However, adequate research is needed to prove whether *Actinobacteria* is related to cellulose degradation. Niu et al. (2015) found that *Tenericutes* were correlated with apparent crude fiber digestibility [61].

At the genus level, *Prevotella*, *Oscillospira*, CF231, *Ruminococcus*, and *Bacteroides* were remarkably enriched in laboratory rats. In particular, *Prevotella* accounts for 20% of the laboratory rats’ gut microbiota, compared with 1.2% of Plateau zokor. *Prevotella* group was relevant to cellulose and xylans digestibility [51,62]. In human beings, *Prevotella*-affiliated readings have been enriched in human metagenomics [54,63]. Native rural Africans who ate more fiber diets showed enrichment of *Prevotella* compared with Africans and Americans who ate more meat and fat [64]. In rumen animals, *Prevotella* [65] is rumen bacteria that have efficiently degrading xylan. In rodents, the cellulolytic activity of pika was positively associated with *Prevotella*, *Ruminococcus*, or CF231, where acetate, butyrate, and total SCFA (Short-chain fatty acid) showed positive correlations with *Prevotella* and CF231 [12]. *Bacteroides bacteria* are typically enriched in herbivores [66]. *Ruminococcus* also show strong correlation with fiber fermentation [67,68], as many members of these genera consist of diverse fibrinolytic and cellulolytic bacterial genera with various cellulase and hemicellulase genes [51,52]. Moreover, *Altererythrobacter* and *Pseudomonas* were more abundant in plateau zokor. However, *Prevotella* shows a negative correlation with *Altererythrobacter* and *Pseudonocardia* in laboratory rats (Figure S1). The gut microbiota associated with cellulose degradation in the intestine of rats is more than that of plateau zokor, which suggest these gut microbiotas might be the compensation for the lightweight and the smaller size of the intestine and large intestine and cecum in laboratory rats. More importantly, these findings were also supported by the functional prediction by the PICRUSt.

## 5. Conclusions

Our findings reveal that the gut microbiota plays an important role in the digestion of plateau zokor. The higher digestibility of crude fiber of plateau zokor than laboratory rats was caused by increasing the length of the cecum and the volume of the cecum tract, as well as gut microbiota associated with cellulose degradation. This study highlights that the gut microbiota and the morphology of the digestive tract are both vital to the digestion of wild animals, and we should consider the factors of animal digestion strategies from multiple angles in the research process. However, there are still shortcomings in this study; the difference of the gut microbiota in the small intestine, large intestine, and cecum of plateau zokor and rats have not been further investigated, and levels of SCFAs and fiber in the feces have not been measured yet. In addition, we should appreciate the functional prediction from 16S sequencing data. In the future, metagenomic sequencing on the contents of different parts of the gut of plateau zokor could be conducted to further explore the role of the gut microbiota in plateau zokor.

## Figures and Tables

**Table 1:** The difference statistical analysis of 16S rRNA clone library between plateau zokors and laboratory rats.

Group	*Myospalax baileyi*	*Rattus norvegicus*	ANOVA (df = 1)		Sample Size
			F	*P*	n1, n2
OTUs	1889.30 ± 262.01	2008.65 ± 294.44	1.046	0.317	11, 12
Chao 1	4010.14 ± 516.92	4380.74 ± 575.65	2.620	0.120	11, 12
Shannon	9.136 ± 0.702	9.266 ± 0.655	0.211	0.650	11, 12
Simpson	0.987 ± 0.013	0.989 ± 0.009	0.365	0.552	11, 12
Good’s coverage	0.829 ± 0.023	0.814 ± 0.027	1.925	0.179	11, 12

Note: n1 represents the sample size of *Myospalax baileyi*, n2 represents the sample size of *Rattus norvegicus*.

**Table 2:** The difference statistical analysis of relative abundance of target gene copies for specific species (% of total bacterial 16SrRNA gene) between plateau zokors and laboratory rats at Phylum level.

Phylum	*Myospalax baileyi*	*Rattus norvegicus*	ANOVA (df = 1)		Sample Size
			F	*P*	n1, n2
*Bacteroidetes*	41.130 ± 9.828	44.094 ± 13.429	0.359	0.555	11, 12
*Firmicutes*	31.672 ± 8.266	36.113 ± 11.663	1.090	0.308	11, 12
*Proteobacteria*	5.963 ± 1.469	4.889 ± 1.686	2.630	0.120	11, 12
*Verrucomicrobia*	4.197 ± 7.290	4.783 ± 6.962	0.039	0.846	11, 12
*Actinobacteria*	3.931 ± 1.078	2.474 ± 0.780	13.961	0.001 **	11, 12
*Planctomycetes*	0.637 ± 0.221	0.714 ± 0.306	0.460	0.505	11, 12
*Acidobacteria*	0.541 ± 0.175	0.426 ± 0.095	3.902	0.062	11, 12
*Chloroflexi*	0.274 ± 0.100	0.248 ± 0.078	0.486	0.493	11 12
*TM7*	0.249 ± 0.230	0.277 ± 0.447	0.033	0.857	11, 12
*Tenericutes*	0.174 ± 0.087	0.731 ± 0.316	31.842	0.000 ***	11, 12
*Gemmatimonadetes*	0.148 ± 0.051	0.119 ± 0.048	3.437	0.078	11, 12

Note: n1 represents the sample size of *Myospalax baileyi*, n2 represents the sample size of *Rattus norvegicus*. The asterisks refer to significant difference (** means *p* < 0.01, *** means *p* < 0.001).

**Table 3:** The difference statistical analysis of relative abundance of target gene copies for specific species (% of total bacterial 16SrRNA gene) between plateau zokors and laboratory rats at Genus level.

Genus	*Myospalax baileyi*	*Rattus norvegicus*	ANOVA (df = 1)		Sample Size
			F	*P*	n1, n2
*Akkermansia*	3.976 ± 7.267	4.498 ± 6.876	0.031	0.861	11, 12
*Lactobacillus*	3.746 ± 5.568	4.458 ± 3.520	0.137	0.715	11, 12
*Prevotella*	1.275 ± 1.563	20.617 ± 14.757	18.630	0.000 ***	11, 12
*Oscillospira*	0.731 ± 0.452	1.962 ± 1.277	9.138	0.006 **	11, 12
*Coprococcus*	0.457 ± 0.530	0.205 ± 0.124	2.552	0.125	11, 12
*Clostridium*	0.387 ± 0.391	0.238 ± 0.119	1.586	0.222	11, 12
*Alicyclobacillus*	0.343 ± 0.102	0.285 ± 0.158	1.065	0.314	11, 12
*Altererythrobacter*	0.283 ± 0.084	0.188 ± 0.072	8.406	0.009 **	11, 12
*Desulfovibrio*	0.236 ± 0.213	0.115 ± 0.115	2.952	0.100	11, 12
*Pseudonocardia*	0.186 ± 0.063	0.137 ± 0.039	5.080	0.035 *	11, 12
*DA101*	0.173 ± 0.062	0.136 ± 0.044	2.725	0.114	11, 12
*Pseudomonas*	0.166 ± 0.064	0.133 ± 0.056	1.698	0.207	11, 12
*Rubrobacter*	0.144 ± 0.057	0.108 ± 0.050	2.574	0.124	11, 12
*Kaistobacter*	0.143 ± 0.043	0.125 ± 0.057	0.699	0.413	11, 12
CF231	0.131 ± 0.138	2.764 ± 2.560	11.543	0.003 **	11, 12
*Ruminococcus*	0.027 ± 0.015	0.177 ± 0.082	34.716	0.000 ***	11, 12
*Bacteroides*	0.018 ± 0.019	1.228 ± 0.916	19.109	0.000 ***	11 12

Note: n1 represents the sample size of *Myospalax baileyi*, n2 represents the sample size of *Rattus norvegicus*. The asterisks refer to significant difference (* means *p* < 0.05, ** means *p* < 0.01, *** means *p* < 0.001).

## Data Availability

Raw reads were submitted to the Sequence Read Archive Database (accession number: SAMN29363577).

## Supplementary Materials

The following supporting information can be downloaded at: https://www.mdpi.com/article/10.3390/ani12162155/s1, Figure S1: The co-occurrence patterns among the 18 core genera across the 11 samples of Plateau zokers (left), and the 20 core genera across the 12 samples of SD rats (right). Co-occurrence patterns were determined by the Spearman’s rank correlation analysis; Table S1: Host species (plateau zokors/laboratory rats) information corresponding to the sample source; Table S2: The nutrient information of Rat Maintenance Feed in the experiment; Table S3: The difference statistical analysis of relative abundance of target gene copies for specific species (% of total bacterial 16SrRNA gene) between plateau zokors and laboratory rats at Class level; Table S4: The difference statistical analysis of relative abundance of target gene copies for specific species (% of total bacterial 16SrRNA gene) between plateau zokors and laboratory rats at Order level; Table S5: The difference statistical analysis of relative abundance of target gene copies for specific species (% of total bacterial 16SrRNA gene) between Plateau zokors and laboratory rats at Family level.
