# Supplementary material for: Digestive Tract Morphology and Gut Microbiota Jointly Determine an Efficient Digestive Strategy in Subterranean Rodents: Plateau Zokor
Source: Animals (Basel). 2022 Aug 22;12(16):2155. doi: 10.3390/ani12162155 (PMC9404879; doi:10.3390/ani12162155)
Supplement: Supplementary file 1 [file animals-12-02155-s001.zip › animals-1813751-supplementary.pdf]

Supplementary Materials

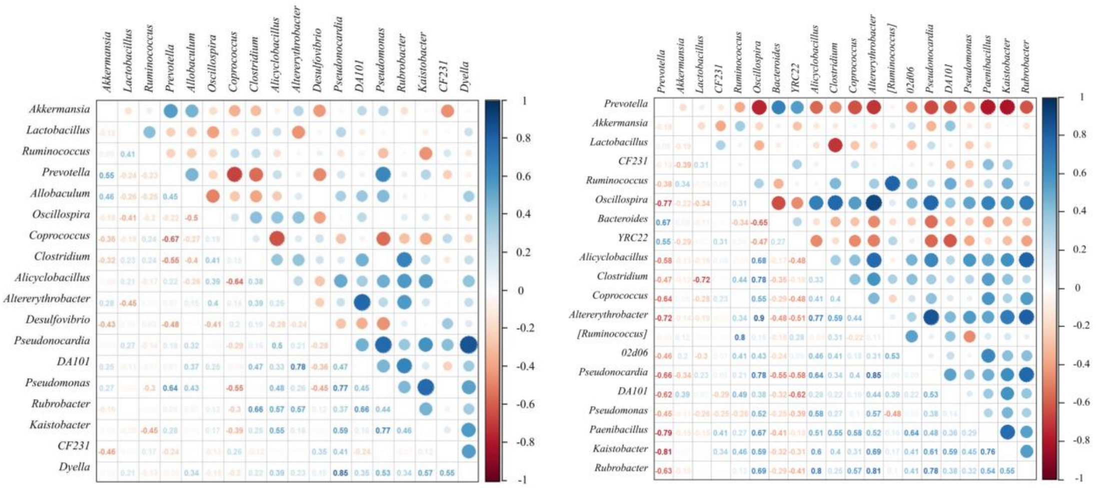

Figure S1. The co-occurrence patterns among the 18 core genera across the 11 samples of Plateau zokers (left), and the 20 core genera across the 12 samples of SD rats (right). Co-occurrence patterns were determined by the Spearman's rank correlation analysis. Correlation coefficient is displayed at the bottom left.

Table S1. Host species (plateau zokors / laboratory rats) information corresponding to the sample source.

Species		ID	Sex	Weight (g)
plateau zokor	*Eospalax baileyi*	Z1	female	216.88
plateau zokor	*Eospalax baileyi*	Z2	male	223.66
plateau zokor	*Eospalax baileyi*	Z3	male	215.09
plateau zokor	*Eospalax baileyi*	Z4	female	229.78
plateau zokor	*Eospalax baileyi*	Z5	female	216.09
plateau zokor	*Eospalax baileyi*	Z6	female	201.24
plateau zokor	*Eospalax baileyi*	Z7	male	222.48
plateau zokor	*Eospalax baileyi*	Z8	female	253.2
plateau zokor	*Eospalax baileyi*	Z9	male	236.86
plateau zokor	*Eospalax baileyi*	Z10	male	235.14
plateau zokor	*Eospalax baileyi*	Z11	female	252.37
plateau zokor	*Eospalax baileyi*	Z12	male	187.95
laboratory rat	*Rattus norvegicus*	R1	male	213.5
laboratory rat	*Rattus norvegicus*	R2	female	195.99
laboratory rat	*Rattus norvegicus*	R3	female	228.41
laboratory rat	*Rattus norvegicus*	R4	female	197.5
laboratory rat	*Rattus norvegicus*	R5	male	196.34
laboratory rat	*Rattus norvegicus*	R6	female	232.82
laboratory rat	*Rattus norvegicus*	R7	female	238.15
laboratory rat	*Rattus norvegicus*	R8	male	216.42
laboratory rat	*Rattus norvegicus*	R9	male	224.18
laboratory rat	*Rattus norvegicus*	R10	male	234.97
laboratory rat	*Rattus norvegicus*	R11	male	238.15
laboratory rat	*Rattus norvegicus*	R12	female	189.04

Note: Host species (plateau zokors / laboratory rats) information corresponding to the sample source. Z01-Z11 means zokor 01-11, SD01-SD12 means laboratory rat 01-11. The weight is the original weight before experiment.

Table S2. The nutrient information of Rat Maintenance Feed in the experiment.

Testitem	Testing date (g / kg)	Sample size	Test reference
Crude protein	221.5±20.0	6	GB/T 6432-1994
Crude fat	43.6±3.0	6	GB/T 6433-2006
Crude fiber	36.9±4.4	6	GB/T 6434-2006
Moisture	81.3±14.8	6	GB/T 6435-2006
Calcium	11.4±0.6	6	GB/T 6436-2002
Phosphorus	8.3±0.8	6	GB/T 6437-2002
Crude ash	68.6±2.4	6	GB/T 6438-2007

Table S3. The difference statistical analysis of relative abundance of target gene copies for specific species (% of total bacterial 16SrRNA gene) between plateau zokors and laboratory rats at Class level.

Class level	*Myospalax baileyi*	*Rattus norvegicus*	ANOVA (df=1)		Sample size
			F	*P*	n1，n2
Actinobacteria	2.673±0.731	1.648±0.488	15.882	0.001	11，12
Flavobacteriia	0.313±0.113	0.197±0.076	8.337	0.009	11，12
4C0d-2	0.061±0.035	0.013±0.015	18.801	0.000	11，12
Nitrospira	0.034±0.020	0.015±0.017	5.685	0.026	11，12
TM7-1	0.057±0.031	0.025±0.017	8.972	0.006	11，12
Mollicutes	0.174±0.087	0.731±0.316	31.841	0.000	11，12
Deinococci	0.010±0.008	0.003±0.006	4.307	0.050	11，12

Note: n1 represents the sample size of *Myospalax baileyi*, n2 represents the sample size of *Rattus norvegicus*.

Table S4. The difference statistical analysis of relative abundance of target gene copies for specific species (% of total bacterial 16SrRNA gene) between plateau zokors and laboratory rats at Order level.

Order level	*Myospalax baileyi*	*Rattus norvegicus*	ANOVA (df=1)		Sample size
			F	*P*	n1，n2
RB41	0.167±0.088	0.107±0.043	4.412	0.047	11，12
Actinomycetales	2.402±0.674	1.499±0.438	14.732	0.001	11，12
Bifidobacteriales	0.082±0.092	0.002±0.005	8.906	0.007	11，12
Flavobacteriales	0.313±0.113	0.197±0.076	8.337	0.008	11，12
YS2	0.061±0.035	0.013±0.015	18.801	0.000	11，12
Nitrospirales	0.034±0.020	0.015±0.017	5.685	0.026	11，12
Enterobacteriales	0.062±0.036	0.031±0.023	6.184	0.021	11，12
PYR10d3	0.104±0.052	0.069±0.023	4.421	0.047	11，12
RF39	0.162±0.086	0.697±0.297	32.976	0.000	11，12
Deinococcales	0.010±0.008	0.003±0.006	4.307	0.050	11，12

Note: n1 represents the sample size of *Myospalax baileyi*, n2 represents the sample size of *Rattus norvegicus.*

Table S5. The difference statistical analysis of relative abundance of target gene copies for specific species (% of total bacterial 16SrRNA gene) between Plateau zokors and laboratory rats at Family level.

Family level	*Myospalax baileyi*	*Rattus norvegicus*	ANOVA (df=1)		Sample size
			F	*P*	n1，n2
Corynebacteriaceae	0.002±0.008	0.051±0.071	4.931	0.037	11，12
Frankiaceae	0.104±0.036	0.060±0.033	9.239	0.006	11，12
Microbacteriaceae	0.207±0.066	0.144±0.057	6.022	0.022	11，12
Pseudonocardiaceae	0.264±0.089	0.184±0.060	6.463	0.018	11，12
Bifidobacteriaceae	0.082±0.092	0.002±0.005	8.906	0.007	11，12
Bacteroidaceae	0.023±0.025	1.236±0.917	19.123	0.000	11，12
Porphyromonadaceae	0.044±0.036	0.137±0.120	6.056	0.022	11，12
Prevotellaceae	1.632±1.529	21.597±14.800	19.675	0.000	11，12
RF16	0.003±0.008	0.089±0.108	6.886	0.015	11，12
S24-7	34.241±8.318	14.005±6.064	45.007	0.000	11，12
[Odoribacteraceae]	0	0.027±0.028	10.639	0.003	11，12
[Paraprevotellaceae]	0.153±0.133	3.218±2.813	12.979	0.001	11，12
Flavobacteriaceae	0.291±0.099	0.179±0.065	10.134	0.004	11，12
Paenibacillaceae	0.054±0.035	0.140±0.122	4.998	0.036	11，12
Planococcaceae	0.060±0.031	0.034±0.017	6.352	0.019	11，12
Turicibacteraceae	0.002±0.005	0.090±0.072	16.344	0.001	11，12
Peptostreptococcaceae	0.022±0.057	0.110±0.080	8.852	0.007	11，12
Ruminococcaceae	5.444±2.172	8.589±2.627	9.679	0.005	11，12
Veillonellaceae	0.010±0.014	0.456±0.446	10.941	0.003	11，12
[Mogibacteriaceae]	0.040±0.033	0.095±0.048	9.554	0.005	11，12
Gemmatimonadaceae	0.008±0.011	0	7.461	0.012	11，12
Nitrospiraceae	0.029±0.020	0.007±0.008	11.448	0.002	11，12
Isosphaeraceae	0.466±0.174	0.328±0.125	4.819	0.039	11，12
Erythrobacteraceae	0.605±0.186	0.440±0.160	5.167	0.033	11，12
Comamonadaceae	0.212±0.083	0.140±0.058	5.799	0.025	11，12
Enterobacteriaceae	0.062±0.036	0.031±0.023	6.184	0.021	11，12
Sinobacteraceae	0.034±0.018	0.021±0.012	4.468	0.046	11，12
Deinococcaceae	0.010±0.008	0.003±0.006	4.307	0.050	11，12

Note: n1 represents the sample size of *Myospalax baileyi*, n2 represents the sample size of *Rattus norvegicus* .
